# Cleaved CDCP1 marks the spot: a neoepitope for RAS-driven cancers

**DOI:** 10.1172/JCI157168

**Published:** 2022-02-15

**Authors:** Katelyn L. Donahue, Marina Pasca di Magliano

**Affiliations:** 1 Graduate Program in Cancer Biology and; 2 Department of Surgery, Department of Cell and Developmental Biology, Rogel Cancer Center, University of Michigan, Ann Arbor, Michigan, USA.

## Abstract

A challenge in cancer treatment is targeting cancer cells while sparing normal cells. Thus, identifying cancer-specific neoepitopes is an active research area. Neoepitopes are generated by the accumulation of mutations; however, deadly cancer types, including pancreatic cancer, have a low mutational burden and, consequently, a paucity of neoantigens. In this issue of the *JCI*, Lim, Zhou, and colleagues describe a neoepitope generated upon proteolytic cleavage of the transmembrane CUB domain containing protein 1 (CDCP1). CDCP1 is overexpressed in cancer and portends a worse prognosis; previous attempts to target CDCP1 reduced cancer growth, but adversely affected the host. Here, the authors generated an antibody that specifically targeted cleaved CDCP1 (c-CDCP1) and developed a drug conjugate, a vector for radioactive ions, and a mediator of T cell activation. The therapeutics inhibited pancreatic cancer cell growth in vitro and in vivo. Exploiting proteolytic cleavage-derived neoantigens opens an attractive way for specifically targeting cancer cells.

## Tumor neoantigens

Engaging the immune system to fight cancer has revolutionized therapy for several malignancies, but has yet to make a positive impact on pancreatic cancer patients (1). Yet studies of long-term survivors have shown lasting immunity directed against tumor antigens (2), indicating that when antitumor immunity does occur, tumor recurrence is prevented. However, it remains a challenge to consistently induce therapeutic antitumor immune responses in most patients with pancreatic cancer. This challenge is due in large part to the mutation burden of pancreatic cancer being relatively low compared with other malignancies (3), thus reducing the potential number of neoantigens. An alternative approach of targeting immune cells to molecules that are overexpressed in cancer, but not mutated, runs a high risk of negatively affecting healthy cells as well. In this issue of the *JCI*, Lim, Zhou, and colleagues identify a tumor neoantigen generated upon cleavage of the CUB domain containing protein 1 (CDCP1) in pancreatic cancer (4). CDCP1, also known as CD318, gp140, SIMA135, and Trask, is a single-pass transmembrane glycoprotein that is overexpressed in several malignancies, including pancreatic cancer, where it correlates with worse prognosis (5).

Functionally, CDCP1 works upstream of Src and PKCδ to promote tumor cell motility and has also been reported to be involved in a variety of other tumorigenic signaling pathways, including EGFR and HIF (6). In pancreatic cancer, the extracellular domain of CDCP1 was previously utilized in a theranostic approach, using a radiolabeled antibody (7) that resulted in a decrease in tumor growth. However, because CDCP1 is expressed by normal cells as well as malignant cells, its therapeutic window is limited. A second approach to targeting CDCP1 in pancreatic cancer involved the development of specific anti-CDCP1 CAR T cells (8). In this study, the authors used flow cytometric analysis of patient-derived xenografts to identify cell-surface targets enriched in pancreatic cancer cells, one of which was CDCP1 (referred to as CD318). The investigators generated CAR T cells specific for CDCP1 and showed efficacy in a transplantation model using human pancreatic cancer cell lines in immunodeficient mice. This previous body of literature supported the notion of targeting CDCP1 in cancer, but raised concerns about targeting a protein that is also present on normal cells, setting the stage for the study by Lim, Zhou, et al. in this issue of the *JCI* (4).

Lim, Zhou, et al. elegantly traversed the breadth of the preclinical discovery process, from identifying key structural elements in CDCP1’s composition to utilizing its cancer-associated neoepitope in therapeutic murine models (4). Proteolysis is upregulated in cancer, and pancreatic cancer is no exception (9). The activation of proteases results in extracellular cleavage of CDCP1. Through a combination of assays designed to explore the nature of a recombinant CDCP1 engineered in-house to include inducible cleavage sites, Lim, Zhou, et al. discerned that the N-terminal fragment (NTF) of CDCP1 surprisingly remained bound to the C-terminal fragment (CTF) of CDCP1 after proteolytic cleavage between these 2 domains. As a result, the structures of full-length and cleaved CDCP1 (fl-CDCP1 and c-CDCP1, respectively) were remarkably similar, as determined using a combination of wet-laboratory techniques and cutting-edge bioinformatic modeling. Other structural observations made to the benefit of both the fundamental biological understanding of CDCP1 and its potential translational applications included the identification of a proteolysis site, termed Cut1 (K365), the finding that the CTF was not expressed without the NTF, and the determination that a single intracellular tyrosine residue, Y734, was essential to the downstream signaling activity of both fl- and c-CDCP1. This comprehensive characterization of the structure and function of fl- and c-CDCP1 culminated in the identification of the c-CDCP1 neoepitope, a cancer-specific target with an abundance of promising therapeutic applications (4).

## Overcoming immunotherapy barriers in pancreatic cancer

**Figure 1:**
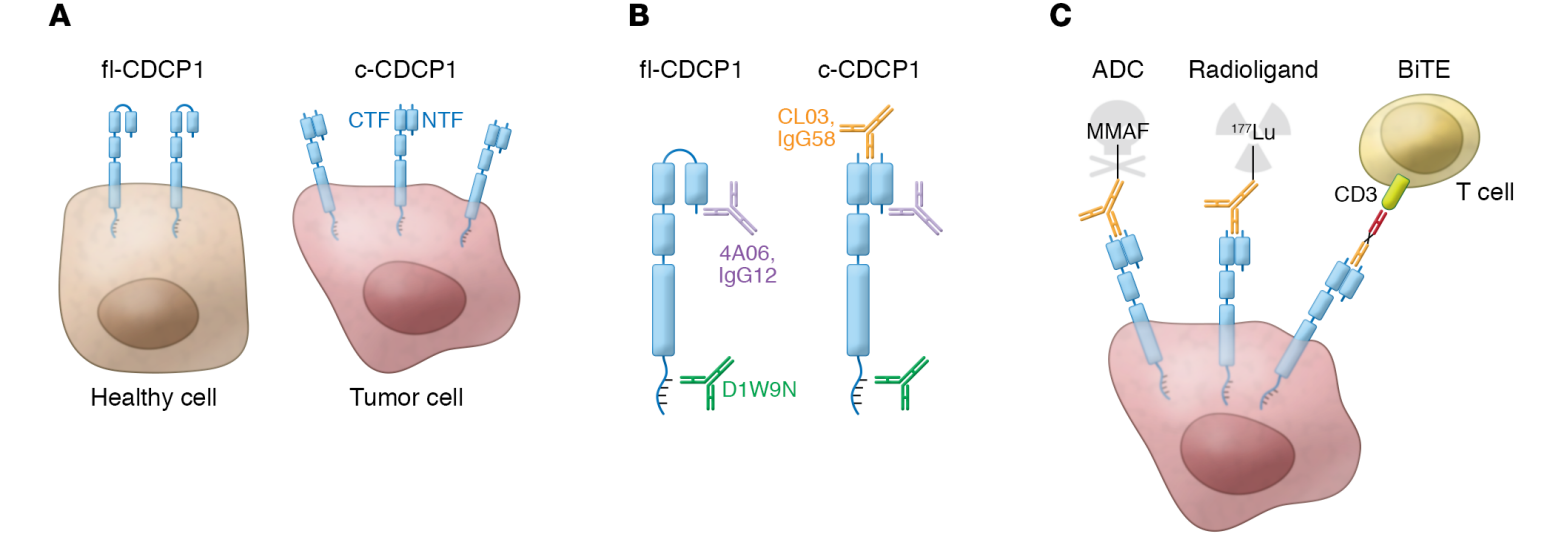
c-CDCP1 marks pancreatic tumor cells for targeted therapeutics. (**A**) Healthy cells express relatively low levels of fl-CDCP1, while pancreatic tumor cells have increased levels of fl-CDCP1 and c-CDCP1. Extracellular proteases cleave CDCP1 between the NTF and CTF. Lim, Zhou, et al. (4) showed that both fragments remain in close association, providing a targetable neoepitope on pancreatic tumor cells. (**B**) Antibodies that bind both fl-CDCP1 and c-CDCP1 include D1W9N (commercially available), which targets the ectodomain; 4A06 (6), which binds the human NTF; and IgG12, (a murine analog of 4A06, developed by Lim, Zhou, et al.), which binds the NTF. Lim, Zhou, et al. also developed two antibodies that bound specifically to c-CDCP1: CL03 (human) and IgG58 (mouse) both bound the neoepitope exposed on the NTF after proteolytic cleavage. (**C**) Lim, Zhou, et al. (4) investigated multiple modes of therapeutic action, assessing the ability of antibodies specific for the c-CDCP1 neoepitope to kill tumor cells while sparing healthy cells. Three strategies included an ADC that used the cytotoxin MMAF, targeted radiation that employed ^177^Lu- IgG58 radioligands, and ligand-dependent T cell activation that created a BiTE molecule through the conjugation of anti-CD3 OKT3 scFV with the c-CDCP1–targeting variable domain.

As posited by Lim, Zhou, et al. (4), the emergence of the c-CDCP1 cancer-associated neoepitope has important therapeutic implications. This neoepitope can be exploited as a mechanism of targeted delivery for small molecules, radiation, and immune activators. At the same time, healthy cells, even if they express CDCP1, have the full-length version and remain unbound by c-CDCP1–specific antibodies. After a comprehensive phage display screening process, two antibodies highly specific for c-CDCP1 over fl-CDCP1 were identified: human clone CL03 and murine clone IgG58. Both clones demonstrated specific targeting of c-CDCP1–bearing tumors in vitro and in vivo; the latter was observed using PET imaging of tumor-bearing mice treated with radiolabeled antibody. While initial observations on the nature of CDCP1 were based on human models, the mouse antibody IgG58 allowed Lim, Zhou, et al. to extend their studies to syngeneic pancreatic cancer models. KPC cells (derived from a commonly used mouse model of pancreatic cancer based on the expression of the hallmark oncogenic mutations in *Kras* and *Trp53*) were transplanted subcutaneously into immunocompetent mice. Impressively, an antibody-drug conjugate (ADC) of CL03 and the cytotoxin monomethyl auristatin F (MMAF) selectively killed c-CDCP1–expressing human tumor cells in vitro, and healthy mice treated with IgG58-MMAF had no evidence of toxicity after 21 days of monitoring. The specificity of c-CDCP1 targeting was investigated further by treating mice harboring subcutaneous c-CDCP1–positive tumors with ^177^Lu-IgG58 radioligand, which dramatically decreased tumor growth and increased survival. The c-CDCP1 antibodies were also shown to work successfully as bispecific T cell engager (BiTE) molecules, as they activated Jurkat T lymphocyte reporter cells in a dose-dependent manner only when cocultured with c-CDCP1–bearing tumor cells (ref. 4 and Figure 1).

The Lim, Zhou, et al. study (4) is highly innovative and presents a potential avenue for overcoming some of the barriers to applying immunotherapy to pancreatic cancer, and will likely lead to clinical studies in the near future. Pancreatic cancer is characterized by an extensive desmoplastic stroma that is rich in extracellular matrix, fibroblasts, and largely suppressive immune cells. CD8^+^ T cells are often excluded from the tumor core and become exhausted when they infiltrate the tumor (10–12). Further, pancreatic cancer is linked to systemic immune suppression (13). A multitude of immune checkpoints are engaged, with substantial heterogeneity across patients. For example, the immune checkpoint TIGIT has recently emerged as an elevated factor in pancreatic cancer that drives immune suppression (14, 15). Combination immunotherapy approaches, targeting multiple components of the innate and adaptive immune system, have resulted in better outcomes in preclinical studies (16), but more work is needed to optimize success for a large portion of patients.

The use of conjugated antibodies in the work by Lim, Zhou, et al. (4) may bypass the need to reactivate the host immune response, but other barriers to an effective immunotherapeutic response will likely persist. The ability of CD8^+^ T cells to infiltrate the tumor and to subsequently overcome exhaustion potentially limits the efficacy of BiTE molecules. Further, targeting a specific cell-surface antigen could result in immunoediting and loss of expression of CDCP1 in cancer cells, potentially leading to tumor relapse over time. Another potential barrier that warrants investigating is whether the antibodies will efficiently penetrate the dense, avascular desmoplastic stroma that is a hallmark of pancreatic cancer (17). Alternatively, combination therapy approaches with agents that disrupt the desmoplastic barriers, such as hyaluronidases (18, 19), could be considered. Trials designed to support the clinical development of these antibodies should include a bedside-to-bench approach, whereby samples collected before and after treatment are evaluated for the emergence of resistance clones and, more importantly, to identify eventual mechanisms of resistance.

## Acknowledgments:

The authors would like to thank members of the Pasca di Magliano laboratory for helpful discussion.

## Version 1 02/15/2022

electronic publication
